# Metformin sensitises hepatocarcinoma cells to methotrexate by targeting dihydrofolate reductase

**DOI:** 10.1038/s41419-021-04199-1

**Published:** 2021-10-02

**Authors:** Yinghui Wang, Hui Lu, Linchong Sun, Xin Chen, Haoran Wei, Caixia Suo, Junru Feng, Mengqiu Yuan, Shengqi Shen, Weidong Jia, Ying Wang, Huafeng Zhang, Zijun Li, Xiuying Zhong, Ping Gao

**Affiliations:** 1 Hefei National Laboratory for Physical Sciences at Microscale, The Chinese Academy of Sciences Key Laboratory of Innate Immunity and Chronic Disease, School of Basic Medical Sciences, Division of Life Science and Medicine, University of Science and Technology of China, Hefei, China; 2 Guangzhou First People’s Hospital, School of Medicine and Institutes for Life Sciences, South China University of Technology, Guangzhou, China; 3 Anhui Key Laboratory of Hepatopancreatobiliary Surgery, Department of General Surgery, Anhui Provincial Hospital, The First Affiliated Hospital of USTC, University of Science and Technology of China, Hefei, Anhui China; 4 Suzhou Hospital of Anhui Medical University, Suzhou, China; 5 Guangdong Provincial Institute of Geriatrics, Guangdong Provincial People’s Hospital, Guangdong Academy of Medical Sciences, Guangzhou, China

**Keywords:** Cancer, Cell biology

## Abstract

Metformin, the first-line drug for type II diabetes, has recently been considered an anticancer agent. However, the molecular target and underlying mechanism of metformin’s anti-cancer effects remain largely unclear. Herein, we report that metformin treatment increases the sensitivity of hepatocarcinoma cells to methotrexate (MTX) by suppressing the expression of the one-carbon metabolism enzyme DHFR. We show that the combination of metformin and MTX blocks nucleotide metabolism and thus effectively inhibits cell cycle progression and tumorigenesis. Mechanistically, metformin not only transcriptionally represses DHFR via E2F4 but also promotes lysosomal degradation of the DHFR protein. Notably, metformin dramatically increases the response of patient-derived hepatocarcinoma organoids to MTX without obvious toxicity to organoids derived from normal liver tissue. Taken together, our findings identify an important role for DHFR in the suppressive effects of metformin on therapeutic resistance, thus revealing a therapeutically targetable potential vulnerability in hepatocarcinoma.

## Introduction

Chemotherapy is one of the most frequently used approaches to treat cancers [1], whereas the resistance to and toxicity of chemotherapeutic agents cause many problems in the clinical optimisation of chemotherapy [2, 3]. Chemotherapeutic resistance is usually induced by long-term exposure to chemotherapeutic drugs and, mechanistically, is caused by the absence or overexpression of molecular targets and the activation of compensatory pathways [3]. Therefore, finding new approaches to manipulate the altered molecular targets or compensatory pathways is an emerging issue related to chemotherapy.

Metformin, the first-line drug for type II diabetes, has been recognised as an anticancer agent in recent years [4–6]. The primary target of metformin in the treatment of type II diabetes was identified as complex I of the electron transport chain in mitochondria which is important for aspartate, purine and pyrimidine synthesis [7–11]. However, the downstream target through which metformin inhibits cancer occurrence and progression is still controversial. Previous studies indicated that in cancer cells, metformin regulates protein translation, autophagy and proliferation via the inhibition of mTOR by activating the pAMPK pathway [12, 13]. However, it was also reported that metformin, independent of AMPK, inhibited mTOR through regulation of the Rag GTPase or REDD1 [14, 15]. Taken together, these works identified a complicated network by which metformin exerts its anticancer effects. Although the AMPK–mTOR axis plays important roles in this network, much about the AMPK-mTOR-independent areas, including specific targets and pathways, remains unknown.

According to a recent study by Yang et al., serine catabolism contributes to NADH accumulation when cell respiration is impaired by metformin, suggesting the potential role of metformin in regulating one-carbon metabolism [16]. Metformin has been reported to inhibit nucleotide synthesis in cancer stem cells [17]. In addition, a study in *C. elegans* indicated that metformin regulates the folate cycle of one-carbon metabolism [18]. Collectively, these studies suggest an association between metformin and one-carbon metabolism, but the underlying mechanism and direct target of metformin are unknown.

Given the anticancer effect of metformin and the relationship between metformin and one-carbon metabolism, we considered the possibility of combining metformin with chemotherapeutic drugs that target one-carbon metabolism. Since methotrexate (MTX), a traditional chemotherapeutic agent, interrupts one-carbon metabolism by inhibiting the synthesis of tetrahydrofolate [19], we considered that it might be a promising drug for combination with metformin in cancer therapy. In this study, we discovered that metformin increased the sensitivity of hepatocarcinoma cells to MTX by downregulating the expression of DHFR, the molecular target of MTX. Combination treatment of metformin and MTX decreased nucleotide metabolism and cell proliferation partially by inhibiting DHFR. Mechanistically, we found that metformin not only transcriptionally inhibits DHFR via E2F4 but also promotes the lysosomal degradation of DHFR, independent of AMPK. Metformin also sensitised hepatocarcinoma cells in mouse and organoid models to MTX by inhibiting DHFR. Taken together, our findings identified DHFR as a novel molecular target of metformin to overcome resistance to MTX and suppress cancer cell proliferation.

## Results

### Metformin sensitises hepatocarcinoma cells to MTX treatment by inhibiting DHFR

**Fig. 1:**
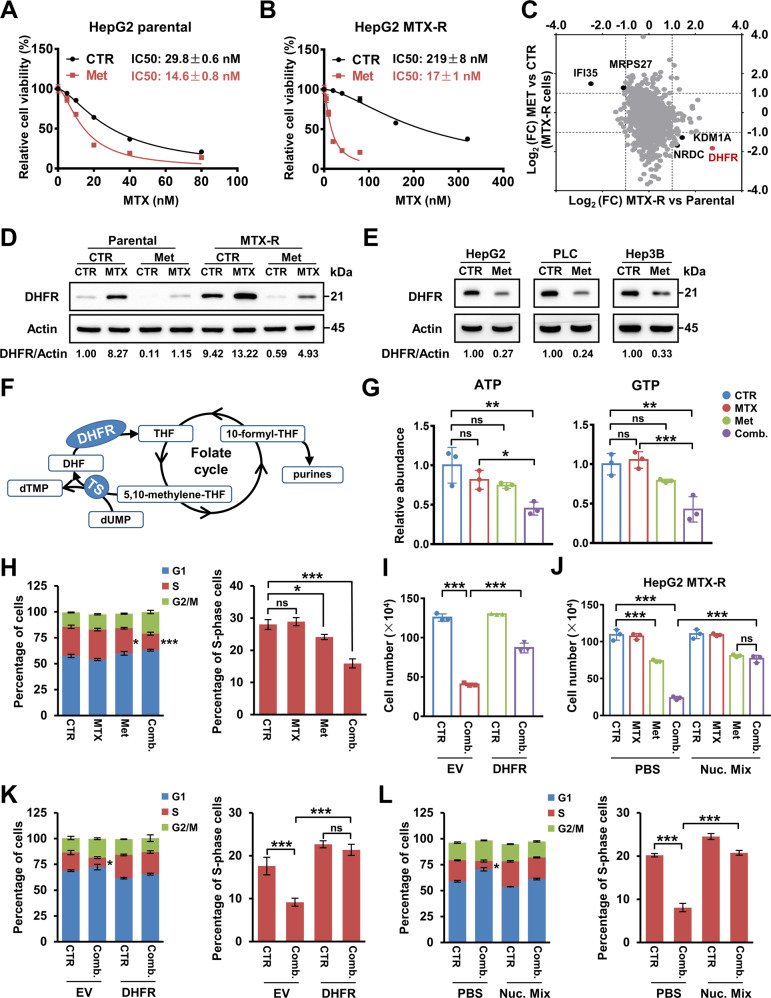
Metformin sensitises hepatocarcinoma cells to MTX treatment by inhibiting DHFR. **A**, **B** Cell numbers of parental (**A**) or MTX-resistant (**B**) HepG2 cells under treatment of different doses of MTX for 72 h with or without metformin (2.5 mM) were measured by a cell counting assay. The IC50 values of MTX in these cells were further calculated with Graphpad Prism 7.0. Data are presented as the mean (±SD) values. **C** Proteomic analysis of protein expression fold changes (FC) between MTX-resistant HepG2 cells and parental HepG2 cells and between MTX-resistant HepG2 cells treated with metformin (2.5 mM) for 48 h and MTX-resistant HepG2 cells treated with PBS. Proteins with significant alterations in MTX-resistant cells that were reversed by metformin are marked in black or red in the diagram. **D** Western blot analysis of DHFR expression in Parental HepG2 and MTX-resistant cells treated with MTX (15 nM), metformin (2.5 mM) or a combination of both for 48 h. **E** Western blot analysis of DHFR expression in HepG2, PLC and Hep3B cells treated with PBS or metformin (2.5 mM) for 48 h. **F** Diagram indicating DHFR’s essential role in purine and pyrimidine synthesis. DHFR catalyses the reduction of dihydrofolate (DHF) to tetrahydrofolate (THF). THF is further transformed into 10-formyl-THF by the folate cycle, which provides 1-C units for purine synthesis. DHFR also associates with thymidine synthase (TS) to generate dTMP from dUMP and 5,10-methylene-THF. **G** MTX-resistant HepG2 cells were treated with MTX (15 nM), metformin (2.5 mM) or a combination of both for 3 days, and the relative abundances of ATP and GTP were measured by LC-MS. **H** The cell cycle in MTX-resistant HepG2 cells treated with MTX (15 nM), metformin (2.5 mM) or a combination of both for 3 days was analysed by flow cytometry. **I** MTX-resistant HepG2 3XFlag-EV and 3XFlag-DHFR cells were treated with DMSO or a combination of MTX (15 nM), metformin (2.5 mM) for 3 days. Cell numbers in the indicated groups were measured by a cell counting assay. **J** MTX-resistant HepG2 cells were treated with MTX (15 nM), metformin (2.5 mM) or a combination of both with or without the addition of a 25 μM nucleotide mixture for 3 days. Cell numbers in the indicated groups were measured by a cell counting assay. **K** Flow cytometric analysis of the cell cycle in MTX-resistant HepG2 3XFlag-EV or 3XFlag-DHFR cells that were treated with DMSO or a combination of MTX (15 nM), metformin (2.5 mM) for 3 days. **L** Flow cytometric analysis of the cell cycle in MTX-resistant HepG2 cells treated with DMSO or a combination of MTX (15 nM), metformin (2.5 mM) metformin with or without the addition of a 25 μM nucleotide mixture for 3 days. Band intensities for protein expressions in the western blot assay were quantitated by ImageJ and normalised to Actin. Data are presented as the mean (±SD) values. Statistical significance was assessed by ANOVA followed by Tukey’s multiple comparisons test. *, **, and *** indicate *P* < 0.05, 0.01, and 0.001, respectively, compared between the indicated groups. ‘ns’ indicates no significant difference between the indicated groups.

To explore the potential role of metformin in disrupting one-carbon metabolism to overcome resistance to MTX, we established MTX-resistant hepatocarcinoma cell lines (HepG2 and PLC) by maintaining parental cells in culture with gradually increasing doses of MTX and assessed the effects of metformin on MTX’s anticancer activity. Interestingly, metformin decreased the IC50 values of MTX in both parental and MTX-resistant cells (Fig. 1A, B and Supplementary Fig. 1A–C), which demonstrated that metformin enhanced the sensitivity of hepatocarcinoma cells to MTX.

We hypothesised that some key proteins responsible for drug resistance in MTX-resistant cells might be affected by metformin treatment. Thus, proteomic analysis of parental HepG2 cells, MTX-resistant HepG2 cells and MTX-resistant HepG2 cells treated with metformin was conducted. As a result, 16 proteins differentially expressed in MTX-resistant cells comparing with parental cells were reversed by metformin treatment (Supplementary Table 1). Intriguingly, DHFR, and KDM1A were highly expressed in MTX-resistant cells compared to the parental cells, while metformin treatment dramatically decreased their expression (Fig. 1C and Supplementary Fig. 1D). And Interferon inducible protein 35 (IFI35) was just the opposite (Fig. 1C). DHFR controls the conversion of DHF to THF, which is crucial to the initiation of the folate cycle. More importantly, DHFR is the original target of MTX. KDM1A, lysine-specific histone demethylase, has been reported to involve in the regulation of cancer stem cells [20]. IFI35 inhibited the proliferation of colorectal cancer cell (CRC) after irradiation, suggesting the potential role of IFI35 in regulating the radiosensitivity of CRC cells [21]. Considering that DHFR is the direct target of MTX and a key enzyme of folate cycle, we focused on DHFR. Western blot analysis also verified the high expression of DHFR in MTX-resistant cells and indicated that metformin reduced the DHFR protein level in both the parental and resistant cells (Fig. 1D and Supplementary Fig. 1E, F). Consistent with previous reports [22, 23], we also observed that MTX treatment increased DHFR protein expression (Fig. 1D and Supplementary Fig. 1E). Interestingly, metformin decreased the DHFR protein level even in the presence of MTX (Fig. 1D and Supplementary Fig. 1E). Furthermore, metformin inhibited DHFR expression in other hepatocarcinoma cell lines, including HepG2, PLC and Hep3B (Fig. 1E). These results suggested the potential role of DHFR in mediating the sensitising effect of metformin.

High expression of DHFR is considered an important cause of MTX resistance [24, 25]. We also found increased expression of DHFR in MTX-resistant cells, and consistent with this finding, forced expression of DHFR in parental cells resulted in resistance to MTX (Fig. 1D and Supplementary Fig. 1E–H). In addition, DHFR knockdown alleviated resistance to MTX in the resistant cells (Supplementary Fig. 1I, J). This evidence further proved the correlation between DHFR expression and MTX resistance, suggesting that metformin reduced MTX resistance via DHFR.

DHFR is essential for the reduction of DHF to THF, thus supporting the synthesis of purines and pyrimidines [26] (Fig. 1F). Indeed, combination treatment of metformin and MTX decreased the ATP and GTP levels in MTX-resistant HepG2 cells (Fig. 1G). Nucleotides are required for cell cycle progression, especially for S phase [27]. We discovered that although MTX alone did not affect the cell cycle progression of MTX-resistant cells, the combination treatment significantly reduced the population of S-phase cells (Fig. 1H and Supplementary Fig. 1K), suggesting that metformin and MTX decreased the nucleotide abundance by inhibiting DHFR, thus interrupting the S phase of the cell cycle and inhibiting cell proliferation. Moreover, restoration of DHFR expression or supplementation with a nucleotide mixture significantly attenuated the suppressive effect on cell numbers and the S-phase population caused by the combination treatment (Fig. 1I–L and Supplementary Fig. 1L–P). Taken together, these results demonstrate that metformin enhances the sensitivity of hepatocarcinoma cells to MTX by inhibiting DHFR and subsequent nucleotide synthesis.

### Metformin transcriptionally suppresses DHFR via E2F4

To determine the mechanism by which metformin downregulates DHFR, we first detected the expression of DHFR in PLC cells treated with pAMPK activator AICAR or the combination of metformin and pAMPK inhibitor compound C, considering the involvement of pAMPK in the anticancer effect of metformin. Neither AICAR nor compound C induced any changes to the regulation of DHFR by metformin (Supplementary Fig. 2A), indicating that pAMPK is not involved in metformin-mediated DHFR expression.

**Fig. 2:**
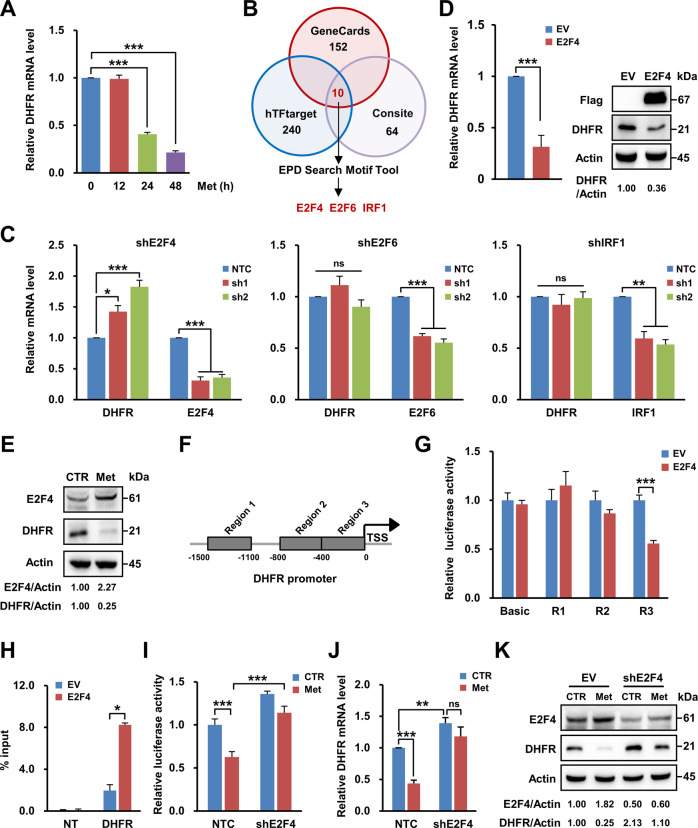
Metformin transcriptionally suppresses DHFR via E2F4. **A** qRT-PCR analysis of DHFR mRNA levels in HepG2 cells treated with metformin (2.5 mM) for the indicated hours. **B** Diagram of the strategy for screening possible transcription factors (TFs) that regulate DHFR. TFs that might bind to the DHFR promoter (−1000 to 0) were collected from TF prediction websites, including GeneCards, hTFtarget and Consite. The common transcription factors that might bind to the DHFR promoter were further validated by the EPD motif tool. **C** qRT-PCR analysis of the mRNA levels of DHFR and E2F4, E2F6 or IRF1 in HepG2 cells expressing NTC shRNA or shRNAs against E2F4 (shE2F4), E2F6 (shE2F6) or IRF1 (shIRF1), respectively. **D** qRT-PCR and western blot analyses of DHFR expression in HepG2 cells expressing 3XFlag-EV or 3XFlag-E2F4. **E** Western blot analysis of the expression of E2F4 and DHFR in HepG2 cells treated with metformin (2.5 mM) for 48 h. **F** A diagram showing the potential E2F4 binding regions in the DHFR promoter. Regions 2 and 3 contain several potential E2F4 binding sites. Region 1 was used as the negative control. **G** A dual-luciferase assay was performed to identify E2F4 binding regions in the DHFR gene. Different regions containing predicted binding sites were inserted into a luciferase reporter vector. E2F4 was co-transfected with pGL3-Basic-Region 1 (R1), pGL3-Basic-Region 2 (R2) or pGL3-Basic-Region 3 (R3). **H** ChIP-qPCR analysis of E2F4 occupancy in the binding region in the DHFR gene in HepG2 cells using IgG or an anti-E2F4 antibody. **I** Luciferase assays were performed to determine the transcriptional activity of DHFR via E2F4 with or without metformin (2.5 mM) treatment. **J** qRT-PCR analysis of DHFR mRNA levels in HepG2 cells expressing NTC shRNA or shRNA against E2F4 (shE2F4) under metformin (2.5 mM) treatment for 24 h. **K** Western blot analysis of the expression of E2F4 and DHFR in HepG2 cells expressing NTC shRNA or shRNA against E2F4 under metformin (2.5 mM) treatment for 48 h. Band intensities for protein expressions in the western blot assay were quantitated by ImageJ and normalised to Actin. Data are presented as the mean (±SEM) or mean (±SD) values. Statistical significance was assessed by Student’s *t*-test or ANOVA followed by Dunnett’s or Tukey’s multiple comparisons test. *, **, and *** indicate *P* < 0.05, 0.01, and 0.001, respectively, compared between the indicated groups. ‘ns’ indicates no significant difference between the indicated groups.

Then, we conducted qRT-PCR analysis and found that metformin significantly decreased the DHFR mRNA level as the treatment time was extended (Fig. 2A and Supplementary Fig. 2B). Analysis of the promoter region of DHFR via transcription factor prediction websites revealed three potential factors—E2F4, IRF1 and E2F6—that might regulate DHFR transcription (Fig. 2B). Further experiments showed that knockdown of IRF1 or E2F6 marginally regulated DHFR transcription; however, knockdown of E2F4 significantly increased the DHFR mRNA level (Fig. 2C). Consistent with this finding, overexpression of E2F4 reduced both the mRNA and protein levels of DHFR (Fig. 2D), indicating that E2F4 inhibits the transcription of DHFR.

Interestingly, E2F4 expression was increased upon metformin treatment in HepG2 cells (Fig. 2E), indicating that metformin might decrease DHFR expression by upregulating E2F4. Bioinformatic analysis of the DHFR gene promoter revealed three potential E2F4 binding regions in the promoter of the DHFR gene (Fig. 2F). The dual-luciferase assay results showed that E2F4 decreased transcription through region 3 of the DHFR promoter rather than through the negative control region (region 1) or another possible binding region (region 2) (Fig. 2G). ChIP-qPCR analysis further proved that E2F4 directly bound to region 3 (Fig. 2H and Supplementary Fig. 2C). Moreover, knockdown of E2F4 reversed the metformin-induced decrease in luciferase activity in 293 cells transfected with plasmids containing region 3 of the DHFR promoter (Fig. 2I). E2F4 knockdown also restored the DHFR mRNA level, which was decreased by metformin (Fig. 2J), demonstrating that metformin inhibited DHFR transcription by upregulating E2F4. However, intriguingly, knockdown of E2F4 did not fully reverse the decrease in the DHFR protein level induced upon metformin treatment (Fig. 2K), implying that other mechanisms might be involved in the inhibition of DHFR by metformin.

### Metformin promotes lysosomal degradation of the DHFR protein

**Fig. 3:**
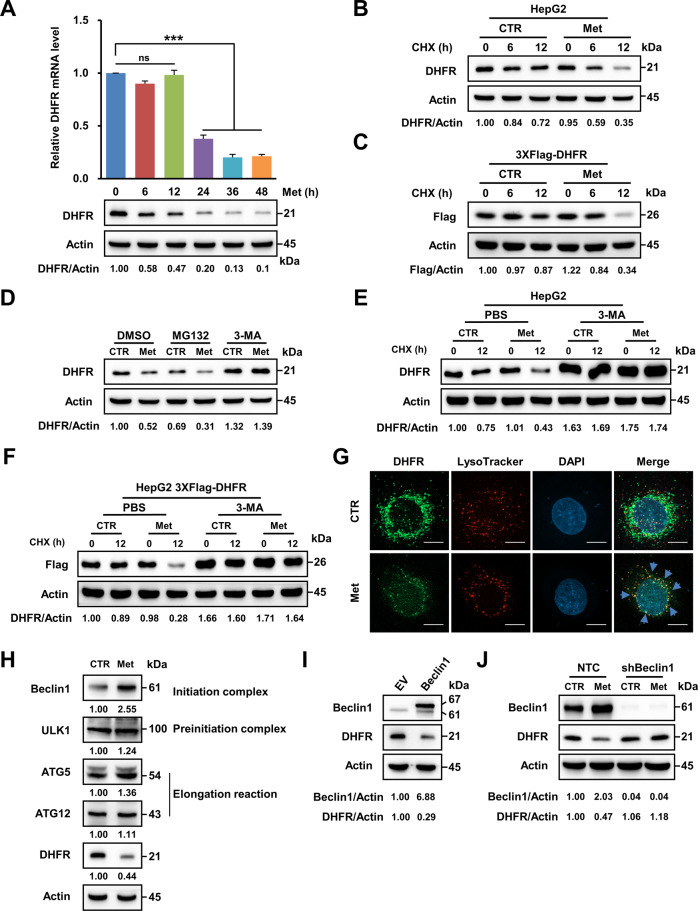
Metformin promotes lysosomal degradation of the DHFR protein. **A** qRT-PCR and western blot analyses of DHFR expression in HepG2 cells under metformin (2.5 mM) treatment for the indicated hours. **B** Western blot analysis of DHFR expression in response to cycloheximide (CHX, 1 µg/mL) with or without metformin (2.5 mM) treatment in HepG2 cells for the indicated hours. **C** Western blot analysis of Flag expression in response to cycloheximide (CHX, 1 µg/mL) in HepG2 cells expressing 3XFlag-DHFR with or without metformin (2.5 mM) treatment. **D** HepG2 cells were treated with PBS, MG132 (5 μM) or 3-MA (1 mM) with or without metformin (2.5 mM) for 12 h. DHFR protein levels were determined by western blot analysis. **E** HepG2 cells were pretreated with PBS or 3-MA (1 mM) for 6 h and then treated with CHX (1 µg/mL), metformin (2.5 mM) or both for 12 h. DHFR protein levels in the indicated cells were then determined by western blot analysis. **F** HepG2 cells overexpressing 3XFlag-DHFR were pretreated with PBS or 3-MA (1 mM) for 6 h and were then treated with CHX (1 µg/mL), metformin (2.5 mM) or both for 12 h. Flag protein levels in the indicated cells were then determined by western blot analysis. **G** PLC cells were treated with or without metformin (2.5 mM) for 24 h, and the medium was then replaced with LysoTracker staining buffer for 2 h. Then the cells were subjected to immunofluorescence assay. DHFR (green), lysosomes (red) and nucleus (blue) were visualised by confocal fluorescence microscopy. Scale bars, 10 µm. **H** Western blot analysis of the expression of autophagy-associated genes and DHFR in HepG2 cells treated with metformin (2.5 mM) for 12 h. Actin was used as the loading control. **I** Western blot analysis of the expression of Beclin1 and DHFR in HepG2 cells overexpressing 3XFlag-EV or 3XFlag-Beclin1. **J** Western blot analysis of the expression of Beclin1 and DHFR in HepG2 cells expressing NTC shRNA or shRNA against Beclin1 (shBeclin1) with or without metformin (2.5 mM) treatment for 12 h. Band intensities for protein expressions in the western blot assay were quantitated by ImageJ and normalised to Actin. Data are presented as the mean (±SEM) of three independent experiments. Statistical significance was assessed by ANOVA followed by Dunnett’s multiple comparisons test. *** indicates *P* < 0.001 compared between the indicated groups. ‘ns’ indicates no significant difference between the indicated groups.

To investigate other pathways that might participate in the inhibition of DHFR by metformin, we measured the changes in DHFR mRNA and protein levels upon metformin treatment at the same time point. The time-course analysis showed that the DHFR mRNA level remained unchanged during the first 12 h; however, the DHFR protein level remarkably decreased during this period (Fig. 3A and Supplementary Fig. 3A), suggesting that metformin might also inhibit DHFR protein expression at early time points.

The rapid decrease in the DHFR protein level was probably due to protein degradation induced by metformin. Thus, we used cycloheximide to block new protein synthesis and measured DHFR protein degradation. We found that metformin accelerated the protein degradation of DHFR in both HepG2 and PLC cells (Fig. 3B and Supplementary Fig. 3B). Furthermore, metformin promoted the degradation of exogenous DHFR protein (Fig. 3C and Supplementary Fig. 3C), verifying that metformin downregulates DHFR by accelerating its protein degradation.

The proteasome pathway and the lysosomal pathway are two major pathways responsible for protein degradation [28]. Our data showed that proteasome pathway inhibitor MG132 did not affect the regulatory effect of metformin on the DHFR protein level, while lysosomal pathway inhibitor 3-MA markedly attenuated the suppressive effect of metformin on DHFR protein expression without affecting DHFR mRNA expression (Fig. 3D and Supplementary Fig. 3D, E). Furthermore, 3-MA pretreatment blocked metformin-induced DHFR protein degradation (Fig. 3E, F and Supplementary Fig. 3F). Confocal imaging showed that the DHFR protein was localised in lysosomes upon metformin treatment (Fig. 3G). Taken together, these data demonstrate that metformin accelerates lysosomal degradation of DHFR in hepatocarcinoma cells.

The formation of autophagosomes requires multiple complexes, including preinitiation, initiation and elongation complexes [29]. While the levels of most proteins in these complexes were slightly affected by metformin, the level of Beclin1, an indispensable molecule in the initiation complex, was obviously increased (Fig. 3H). Furthermore, overexpression of Beclin1 remarkably decreased the DHFR protein level without affecting its mRNA level (Fig. 3I and Supplementary Fig. 3G). More importantly, knockdown of Beclin1 abolished the suppressive effect of metformin on DHFR protein expression (Fig. 3J), indicating that metformin decreased the DHFR protein level through Beclin1-mediated lysosomal degradation.

### DHFR is important for the suppressive effects of metformin on MTX resistance in cancer cells in vivo

**Fig. 4:**
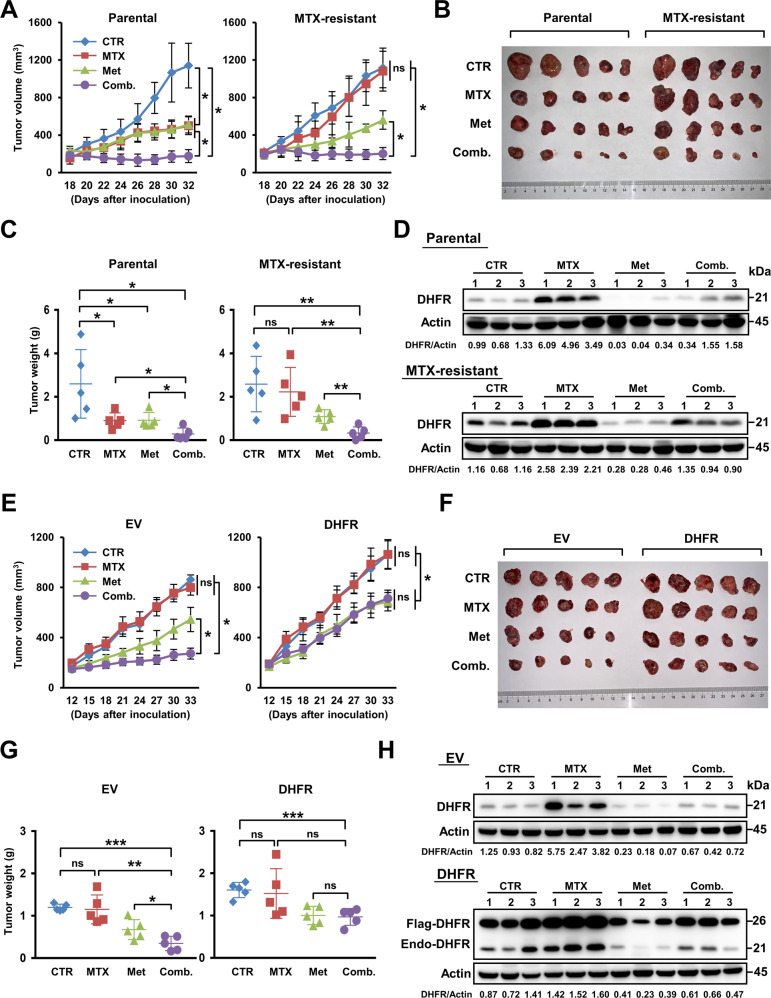
DHFR is important for the suppressive effects of metformin on MTX resistance in cancer cells in vivo. **A**–**D** Male BALB/c nude mice were implanted subcutaneously with Parental HepG2 cells or MTX-resistant HepG2 cells in the flank. After the average tumour volume reached ~200 mm^3^, mice were treated with an i.p. injection of saline or MTX (10 mg/kg) every 4 days and intragastric administration of water or metformin (160 mg/kg) every other day (*n* = 5 per group). **A** Tumour volume was determined based on calliper measurements every 2 days from days 18 to 32. **B**, **C** Tumours described in **A** were excised on day 33. Tumour volume (**B**) and tumour weight (**C**) were compared between the indicated groups. **D** Western blot analysis of DHFR expression using lysates of tumour tissues described in **B**. Actin was used as the loading control. **E**–**H** Male BALB/c nude mice were implanted subcutaneously with MTX-resistant HepG2 EV cells or MTX-resistant HepG2 DHFR cells in the flank. After the average tumour volume reached ~200 mm^3^, mice were treated with an i.p. injection of saline or MTX (10 mg/kg) every 4 days and intragastric administration of water or metformin (160 mg/kg) every other day (*n* = 5 per group). **E** Tumour volume was determined based on calliper measurements every 3 days from days 12 to 33. **F**–**G** Tumours described in **E** were excised on day 34. Tumour volume (**F**) and tumour weight (**G**) were compared between the indicated groups. **H** Western blot analysis of DHFR expression using lysates of tumour tissues described in **F**. Actin was used as the loading control. endo-DHFR: endogenous DHFR. Band intensities for protein expressions in the western blot assay were quantitated by ImageJ and normalised to Actin. Data are presented as the mean (±SEM) or mean (±SD) values. Statistical significance was assessed by ANOVA followed by Tukey’s multiple comparisons test or non-parametric test. *, **, and *** indicate *P* < 0.05, 0.01, and 0.001, respectively, compared between the indicated groups. ‘ns’ indicates no significant difference between the indicated groups.

To further evaluate the effect of metformin on DHFR expression and tumour cell sensitivity to MTX in vivo, xenograft experiments in nude mice were conducted. Interestingly, compared with metformin or MTX treatment alone, the combination of metformin and MTX dramatically inhibited tumour growth even in the MTX-resistant group (Fig. 4A–C), suggesting that metformin enhanced the sensitivity of hepatocarcinoma cells to MTX in vivo. Moreover, metformin treatment had no effect on mouse body weight (Supplementary Fig. 4A), suggesting the low toxicity of metformin in vivo. Western blot analysis confirmed that metformin decreased the DHFR protein level in both groups (Fig. 4D), indicating the important role of DHFR in the anticancer effects of metformin. To further assess the toxicity of the combination of metformin and MTX, C57B6 xenograft model was employed. Consistently, the combination treatment significantly reduced the tumour volume and tumour weight compared with the CTR group (Supplementary Fig. 4B–D). The DHFR protein level was also decreased by metformin (Supplementary Fig. 4E). More importantly, mouse body weight and blood tests did not show significant differences among these four groups (Supplementary Fig. 4F–H), indicating that the toxicity of the combination treatment was relatively low.

To further prove the role of DHFR in the anticancer effects of metformin in vivo, we conducted xenograft experiments using two additional resistant cell lines that stably expressed 3XFlag-EV or 3XFlag-DHFR (Supplementary Fig. 4I). Compared with MTX or metformin treatment alone, the combination treatment significantly inhibited tumour growth in the MTX-resistant EV group (Fig. 4E–G). However, forced overexpression of DHFR abolished the sensitising effect of metformin (Fig. 4E–G), further proving that DHFR is important for metformin-mediated sensitisation of cancer cells to MTX. Again, these treatments showed no obvious effect on mouse body weight (Supplementary Fig. 4J). Western blot analysis confirmed the expression of DHFR in the tumour tissues (Fig. 4H). In summary, these xenograft experiments demonstrated that metformin sensitises hepatocarcinoma cells to MTX via DHFR.

### Metformin enhances the sensitivity of patient-derived organoids to MTX by decreasing the DHFR level

**Fig. 5:**
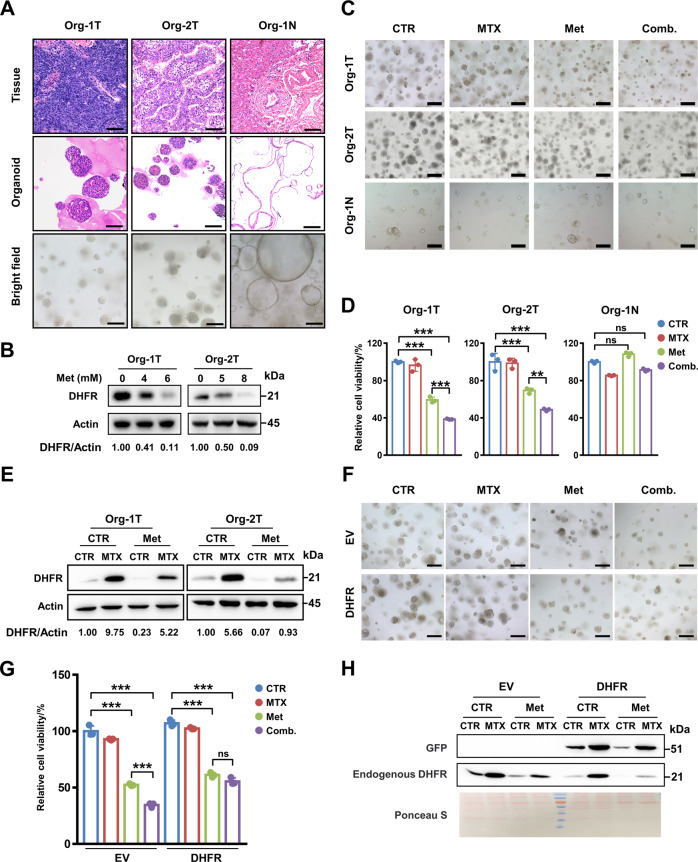
Metformin enhances sensitivity to MTX in patient-derived organoids by decreasing the DHFR level. **A** H&E staining of original tissue and organoids; bright-field images of Org-1T, Org-2T and Org-1N. Scale bar, 50 μm for H&E staining images and 100 μm for bright field images. **B** Western blot analysis of DHFR expression in Org-1T and Org-2T under treatment with different doses of metformin as indicated. **C**–**E** Org-1T, Org-2T and Org-1N were treated with DMSO, MTX (15 nM for Org-1T and 30 nM for Org-2T and Org-1N), metformin (4 mM for Org-1T and 5 mM for Org-2T and Org-1N) or a combination of MTX and metformin for 6 days. Then, representative bright-field images (**C**), relative cell viability (**D**) and DHFR expression in Org-1T and Org-2T (**E**) were analysed. Org-1N was differentiated into hepatocytes before treatment. Scale bar, 100 μm. **F**–**H** Org-1T expressing GFP-EV or GFP-DHFR was treated with DMSO, MTX (15 nM), metformin (4 mM) or a combination of MTX and metformin for 6 days. Then, representative bright-field images (**F**), relative cell viability (**G**) and expression levels of GFP and endogenous DHFR (**H**) were analysed. Scale bar, 100 μm. Ponceau S staining was used as the loading control. Band intensities for protein expressions in the western blot assay were quantitated by ImageJ and normalised to Actin. Data are presented as the mean (±SD) values. Statistical significance was assessed by ANOVA followed by Tukey’s multiple comparisons test or non-parametric test. ** and *** indicate *P* < 0.01 and 0.001, respectively, compared between the indicated groups. ‘ns’ indicates no significant difference between the indicated groups.

To further explore the potential clinical significance of combination therapy with metformin and MTX, a patient-derived organoid model of hepatocarcinoma was employed. We established two tumouroids named as Org-1T and Org-2T from HCC tissues and a normal liver organoid, Org-1N, from liver progenitor cells in healthy liver tissue from a liver haemangioma patient (Fig. 5A). Consistent with previous reports [30–32], Org-1T and Org-2T formed compact spheroid morphology, while Org-1N grew as a lumen structure (Fig. 5A). To evaluate the toxicity of metformin and MTX in hepatocytes, we differentiated liver organoids into hepatocytes using a differentiation medium (DM). qRT-PCR analysis showed increased expression of the classical hepatocyte markers ALB, CYP3A4 and CYP1A2 and decreased expression of the ductal marker SOX9 after DM treatment (Supplementary Fig. 5A), confirming successful hepatocyte differentiation. The IC50 values of metformin and MTX in Org-1T and Org-2T were then measured by Cell Titer-Glo assay (Supplementary Fig. 5B, C). Importantly, western blot analysis proved that metformin inhibited DHFR protein expression in both tumouroids (Fig. 5B).

Interestingly, the combination of MTX and metformin decreased the viability of both Org-1T and Org-2T comparing with MTX or metformin treatment alone (Fig. 5C, D). However, the viabilities of Org-1N remained almost unchanged in these four groups (Fig. 5C, D), suggesting that the combination treatment exhibits low toxicity in normal tissues. More importantly, MTX alone induced DHFR protein expression, which was decreased by metformin, in both Org-1T and Org-2T cells (Fig. 5E). These results verified that metformin effectively decreased DHFR expression and increased MTX sensitivity in a patient-derived organoid model that was similar to the clinical physiological conditions.

We further evaluated the inhibitory effect of the combination treatment with metformin and MTX on Org-1T expressing GFP-DHFR. Comparing with the strong viability inhibition of the EV group, combination treatment only exhibited an inhibitory effect similar to that of metformin treatment alone in DHFR overexpressing Org-1T (Fig. 5F, G). Western blot analysis confirmed the decrease in DHFR expression induced by metformin (Fig. 5H). Collectively, these data verified that decreased DHFR expression is important for metformin-mediated sensitisation of patient-derived organoids to MTX.

## Discussion

**Fig. 6:**
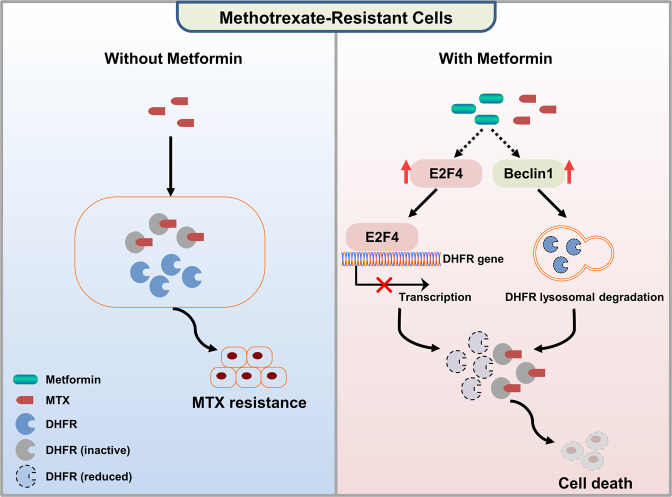
Metformin sensitises hepatocarcinoma cells to MTX by targeting DHFR through both transcriptional and posttranslational mechanisms. Schematic showing that MTX sensitises hepatocarcinoma cells to MTX by targeting DHFR. MTX resistance is usually caused by the high expression of DHFR protein. The combination of metformin and MTX efficiently inhibited hepatocarcinoma cells by decreasing the DHFR level. Mechanistically, metformin not only transcriptionally represses DHFR via E2F4 but also promotes Beclin1-mediated lysosomal degradation of the DHFR protein, thus overcoming the MTX resistance caused by highly expressed DHFR protein.

Historically, a major obstacle in clinical cancer therapy has been the limited success rate of chemotherapy because of the resistance to and toxicity of chemotherapeutic drugs. In this study, we explored whether the recently recognised anticancer agent metformin could be beneficial for solving this problem with chemotherapy, especially for chemotherapy with agents targeting one-carbon metabolism. We demonstrated that metformin inhibited a novel target, DHFR, through both transcriptional and posttranslational mechanisms, thus increasing the sensitivity of hepatocarcinoma cells to MTX and suppressing their proliferation (Fig. 6).

MTX is a traditional chemotherapeutic drug that targets the key enzyme DHFR in one-carbon metabolism to inhibit tumour growth. However, high expression of DHFR is always correlated with MTX resistance in many tumours. Combination therapies are usually employed to solve chemotherapy-related problems. Metformin, which has been considered an anticancer agent in recent years, has little toxicity or side effects in the human body, making it a promising adjuvant agent for combination therapy. Indeed, our data revealed that metformin increased the sensitivity of hepatocarcinoma cells to MTX both in vitro and in vivo (Figs. 1A, B and 4A–C). Furthermore, metformin alone or in combination with MTX exhibited no effect on mouse body weight and caused no obvious illness (Supplementary Fig. 4), suggesting that metformin also ameliorates the toxicity of traditional drugs. Phenformin, a biguanide similar to metformin, has recently been reported to improve the chemotherapeutic response of MTX in breast cancer cells [33]. These findings proved that the combination of metformin and MTX is a potential combination strategy for overcoming chemotherapeutic resistance in the clinic.

Although the primary target of metformin in type II diabetes was identified as complex I of the ETC in mitochondria, the specific target of metformin in cancer cells is still controversial. In this study, we conducted proteomic analysis and uncovered a novel target of metformin, DHFR, whose expression was dramatically decreased by metformin in multiple hepatocarcinoma cell lines (Fig. 1C–E). Intriguingly, this decrease in DHFR expression by metformin was independent of AMPK (Supplementary Fig. 2A). Instead, metformin inhibited DHFR mRNA expression by upregulating the repressive transcription factor E2F4 (Fig. 2). Furthermore, we discovered that the lysosomal degradation of DHFR protein was promoted by metformin treatment (Fig. 3 and Supplementary Fig. 3). DHFR was supposedly degraded in lysosomes through chaperone-mediated autophagy (CMA) according to a previous study [34]. However, lysosomal degradation of DHFR under metformin treatment was attenuated by both 3-MA, an inhibitor of macroautophagy, and shRNAs targeting Beclin1, a key regulator of the autophagosomes (Fig. 3D–J), suggesting the promotive effect of metformin on lysosomal degradation of DHFR was likely caused by autophagy rather than CMA, thus revealing a new mechanism of DHFR protein degradation. Future studies along this line are warranted to provide more mechanistic details regarding the functions of metformin for cancer therapy.

The assessment of combination therapy in vitro is highly insufficient and inaccurate. Organoids are currently widely used in studies of developmental biology and diseases because they maintain structures and functions similar to the originating tissue, which are suitable for the assessment of combination therapies. Excitingly, metformin also increased the sensitivity of patient-derived hepatocarcinoma organoids to MTX while exhibited low toxicity in the organoid models (Fig. 5C, D), further implying the clinical significance of the sensitising effect of metformin. Taken together, our study demonstrated that treatment with metformin effectively sensitised hepatocarcinoma cells to MTX by inhibiting DHFR, which sheds new light on the anticancer effects of metformin and its clinical use for hepatocarcinoma chemotherapy.

## Materials and methods

### Cell culture and reagents

Human hepatocarcinoma cells (HepG2, PLC and Hep3B) were purchased from the ATCC and cultured in DMEM supplemented with 10% FBS and 1% penicillin–streptomycin. All cell lines were tested for Mycoplasma contamination and no cell lines were contaminated. Metformin (HY-17471A), CHX (HY-12320), 3-MA (HY-19312), and PI (HY-D0815) were purchased from MCE (China). Methotrexate (PHR1396), AICAR (A9978) and MG132 (M8699) were purchased from Sigma Aldrich (USA). Compound C (sc-200689) was purchased from Santa Cruz (USA).

### Establishment of resistant cell lines

MTX-resistant HepG2 and PLC cell lines were generated through screening by incubation with gradually increasing doses of MTX. The IC50 of resistant cell lines was determined by an MTT assay, and the resistance index (RI) was further calculated as the IC50(resistant cells)/IC50(parental cells). RI > 5 was considered to indicate effective resistance to MTX. Resistant cells were maintained in complete DMEM containing MTX at a concentration 2× the IC50 of resistant cells.

### MTT assay and calculation of IC50

Cells were incubated in 96-well plates with different doses of metformin or MTX for 72 h. Thereafter, MTT (Sigma Aldrich) was added to the medium for another 4 h. Then, solubilizing buffer (10% w/v SDS, 1% v/v isobutyl alcohol, and 0.1% v/v 10 M HCl) was added to the medium overnight. The OD570 was further determined using a Biotek Cytation 5 microplate reader (USA).

IC50 was calculated based on the results of MTT assay or cell counting assay with Graphpad Prism 7.0. Equation ‘[Inhibitor] vs. normalised response – Variable slop’ in XY analyses ‘Nonlinear regression (curve fit)’ with ‘Least squares (ordinary) fit’ as the fitting method was applied to calculate the IC50 of MTX or metformin.

### Proteomic analysis

Proteomic analysis was conducted according to protocols in previous reports [35–37]. Briefly, cells were collected by trypsin digestion and were then lysed in RIPA buffer. After quantification by the Bradford assay, each aliquot of 250 μg of protein was diluted in 300 μL of UA buffer (8 M urea in 0.1 M Tris-HCl, pH 8.5). Then, the sample was enriched in a 10 K MWCO concentrator (Thermo, USA), alkylated with iodoacetamide (IAA) solution (50 mM IAA in UA buffer) and washed with 50 mM NH4HCO3. Next, the sample was digested in 100 μL of 50 mM NH4HCO3 containing trypsin with a trypsin (Thermo): total protein ratio of ~1:50 in the sample at 37 °C overnight. The peptides were collected and washed twice with 50 μL of NH4HCO3 by centrifugation in a new tube. The flow-through fractions were acidified with TFA at a final concentration of 0.4%. The peptides were then captured, concentrated, desalted and eluted with C18 tips (Thermo), dried with a centrifugal evaporation concentrator and resolved in MS-grade water.

The peptides prepared as described above were then analysed using a Q Exactive Plus mass spectrometer (Thermo) coupled to an EASY-nLC 1200 HPLC system (Thermo) via a nano-electrospray ion source in data-dependent mode. The raw mass spectrometry (MS) data were searched against the human UniProt database and further analysed using Proteomics Discovery Software (version 2.1, Thermo Fisher Scientific). The raw data was provided in Supplementary Table 2.

Logarithms of the fold change ‘MTX-resistant vs parental’ and ‘MTX-resistant Met vs MTX-resistant CTR’ were further analysed by the four-quadrant diagram. The threshold of fold change was set to 2 and 0.5. The proteins in the 4th quadrant and 2nd quadrant that were oppositely regulated by MTX resistance and metformin were both listed in Supplementary Table 1.

### Targeted liquid-chromatography mass spectrometry (LC/MS)

Cells grown in 10-cm tissue culture dishes were washed twice with PBS and lysed in 80% (vol/vol) methanol at −78 °C to extract intracellular metabolites. Cell debris was removed by centrifugation at 4 °C. The supernatant containing metabolites was dried under N2 and then were resuspended using 80 µL LC/MS-grade water. Samples were injected and analysed using AB Triple TOF 5600 plus mass spectrometer (AB/SCIEX, USA) coupled to an HPLC system (AB/SCIEX). ATP and GTP were measured as described in previous studies [17, 38]. Peaks representing each metabolite were extracted and analysed using MultiQuant software.

### Cell cycle analysis

Cells were harvested by trypsin digestion, washed twice with PBS containing 5% FBS, fixed in 70% ethanol, and stained with 20 µg/mL propidium iodide containing 20 µg/mL RNase (DNase-free). Stained cells were analysed by flow cytometry.

### Western blotting

Cells were lysed with RIPA buffer, and equal amounts of protein lysate were boiled and fractionated by 7–10% SDS-PAGE. Primary antibodies against DHFR, Actin, Flag, E2F4, Beclin1, ULK1, ATG5, ATG12 (all from Proteintech, USA) and pAMPK (from Cell Signaling Technology, USA) were then used, and signals were detected using Western ECL Substrate (Bio-Rad, USA or Tanon, China). The detailed information on the antibodies was provided in Supplementary Table 3. The western blotting experiments were repeated three times and the representative results were shown in the final figures. Band intensities of indicated blots were quantitated by ImageJ and were normalised to the intensities of the corresponding Actin.

### RNA extraction and qRT-PCR

Total RNA was isolated using TRIzol and was then subjected to DNase (Ambion, USA) treatment and reverse transcription with a HiScript II 1st Strand cDNA Synthesis Kit (Vazyme, China). qRT-PCR was then performed using SYBR Green Master Mix (Vazyme) in a Roche LightCycler 96 instrument (Switzerland). Primer sequences are listed in Supplementary Table 3. All samples were normalised to 18S rRNA.

### Plasmids and establishment of stable cell lines

shRNAs in the PLKO.1 vector against DHFR, E2F4, E2F6, IRF1 and Beclin1 were purchased from Sigma Aldrich. The coding sequences of human DHFR, E2F4 and Beclin1 were sub-cloned into the pSin or pCDH lentiviral vectors (with a 3XFlag tag or GFP tag). Transduction and viral infection were performed as previously described [39].

### Luciferase assay

Regions 1, 2 and 3 of the DHFR promoter were inserted separately into the pGL3-Basic dual-luciferase reporter vector, and the constructs were designated pGL3-Region 1, Region 2 and Region 3. HEK293 cells in a 48-well plate were co-transfected with either pGL3-Basic-EV or pGL3-Region 1, pGL3-Region 2 or pGL3-Region 3, along with pCDH-3XFlag-EV or pCDH-3XFlag-E2F4, using Lipofectamine 2000 (Invitrogen, USA). Luciferase activity was measured 48 h after transfection using the Dual-Luciferase Reporter Assay System (Promega, USA). Firefly luciferase activity was normalised to Renilla luciferase activity.

### ChIP assay

The ChIP assay was performed with an EZ-ChIP kit (Millipore, USA) following the manufacturer’s instructions. Briefly, cells were fixed with 1% formaldehyde and quenched in 0.125 M glycine. Cells were sonicated in a Bioruptor Sonication System UCD-300. DNA was immunoprecipitated with either control IgG or an anti-E2F4 primary antibody. RNA and protein were digested using RNase A and Proteinase K, respectively, prior to qRT-PCR analysis using SYBR Green Master Mix (Vazyme). Oligos used in this analysis are listed in Supplementary Table 3.

### Immunofluorescence assay

To visualise the colocalization of the DHFR protein and lysosomes, PLC cells were treated with or without metformin (2.5 mM) for 24 h, and the medium was then replaced with LysoTracker staining buffer (Beyotime, China) for 2 h. Then the cells were washed by PBS twice, fixed in 4% paraformaldehyde, blocked in 2% bovine serum albumin in PBS for 1 h, and incubated with DHFR antibody (1:100) overnight. Then the cells were incubated with Coralite488-conjugated secondary antibody (Proteintech, 1:200) for 2 h, and stained with DAPI (Beyotime) for 10 min. Immunofluorescence images were obtained and analysed using a Zeiss LSM800 microscope and Zen blue software.

### Animal study

For xenograft experiments in nude mice, 4 × 10^6^ Parental HepG2 cells, MTX-resistant HepG2 cells, HepG2 MTX-R-EV cells or HepG2 MTX-R-DHFR cells were injected subcutaneously into 6-week-old male nude mice (SJA Laboratory Animal Company, China). For xenograft experiments in C57B6, 5 × 10^6^ Hepa1-6 cells were injected subcutaneously into 6-week-old male C57B6 mice (SJA Laboratory Animal Company, China). After the tumour volume reached ~150–200 mm^3^, the mice were mixed into one cage and randomised into four groups and were treated with an i.p. injection of saline or MTX (10 mg/kg) every 4 days and intragastric administration of water or metformin (160 mg/kg) every other day (*n* = 5 per group). Tumours were measured using digital callipers, and volumes were then calculated using the following equation: length (mm) × width (mm) × depth (mm) × 0.5236. Blood tests of the whole blood (including haemoglobin, platelets, white blood cells and neutrophils) were conducted using a Mindray blood cell analyzer (BC-5000 Vet, China). Enzyme activities of ALT and AST and concentrations of UREA in the serum were measured by a HITACHI biochemical automatic analyzer (3100 Automatic Analyzer, Japan) using kits from Shanghai Kehua Bio-engineering (China).

### Isolation and culture of organoids from healthy human liver and liver tumour tissue

Healthy liver organoids were derived from the non-tumour liver tissue of hepatic haemangioma patients, whereas tumouroids were derived from the tumour tissue of HCC patients. The information of patients was provided in Supplementary Table 4. All of these organoids were cultured according to previously described protocols [31, 40] with slight modifications. Briefly, tissue (~0.25–1 cm^3^) was minced and digested with 2.5 mg/mL collagenase D (Roche) and 0.1 mg/mL DNase (Sigma) at 37 °C with digestion solution. Incubation was performed for 30 min to 1 h for healthy liver tissue. For tumour tissue, to reduce contamination by duct cells, which can be cultured to form normal liver organoids, digestion was continued for 2–3 h. After digestion, the suspensions of normal liver tissue and tumour tissue were then filtered through a 70- or 100-µm nylon cell strainer and spun at 400×*g* for 5 min at 8 °C. Pellets were washed in cold PBS and were then mixed with BME. A total of 2000–5000 cells were seeded per well in a 24-well multiwell plate. After the BME had solidified, cells obtained from healthy liver tissue were cultured in classical human liver organoid isolation medium (Advanced DMEM/F12 supplemented with 1% penicillin/streptomycin, 1% Glutamax, 10 mM HEPES, 1:50 B27 supplement (without vitamin A), 1:100 N2 supplement, 1.25 mM *N*-acetyl-l-cysteine, 10% (v/v) Rspo-1 conditioned medium, 30% (v/v) Wnt3a-conditioned medium, 10 mM nicotinamide, 10 nM recombinant human (Leu15)-gastrin I, 50 ng/mL recombinant human EGF, 100 ng/mL recombinant human FGF10, 25 ng/mL recombinant human HGF, 10 µM forskolin, 5 µM A83-01, 25 ng/mL Noggin and 10 µM Y27632, as described before [40]). Tumour cells were cultured in tumouroid-specific isolation medium (classical human liver organoid isolation medium without Noggin, Rspo-1 and Wnt3a-conditioned medium but supplemented with 3 nM dexamethasone). Liver organoids were passaged by mechanical dissociation using a 1000-µL pipette or briefly digested with 0.25% trypsin–EDTA. Tumouroids were passaged by digestion with 0.25% trypsin–EDTA. After the first passage, liver organoids were cultured in an expansion medium (classical human liver organoid isolation medium without Noggin, Wnt3a, Y27632).

### In vitro hepatocyte differentiation

Liver organoids were cultured in an expansion medium supplemented with 25 ng/mL BMP7 for 5 days. Then, the medium was replaced with differentiation medium (Advanced DMEM/F12 supplemented with 1% penicillin/streptomycin, 1% Glutamax, 10 mM HEPES, 1:50 B27 supplement (with vitamin A), 1:100 N2 supplement, 1 mM N-acetylcysteine, 10 nM recombinant human [Leu15]-gastrin I, 50 ng/mL recombinant human EGF, 25 ng/mL recombinant human HGF, 0.5 µM A83-01, 10 µM DAPT, 3 µM dexamethasone, 25 ng/mL BMP7 and 100 ng/mL recombinant human FGF19) for 10 days, and the medium was changed every 3 days.

### H&E staining

Tissues and organoids were fixed for at least 24 and 0.5 h, respectively, in 10% neutral-buffered formalin (Solarbio, China) at room temperature and were then processed through standard procedures for embedding in paraffin. Briefly, tissues were processed through a graded ethanol series and then immersed in xylene. Organoids were dehydrated by three incubation steps in 100% ethanol for 30 min each. It is important to pre-dye organoids using eosin before or during dehydration. After three washes with xylene for 30 min each and incubation with paraffin overnight, organoids were embedded in paraffin. For HE staining, paraffin sections were cut at a thickness of 4 µm and were then stained following standard procedures.

### Drug treatment of organoids

HCC organoids and differentiated liver organoids were digested into single cells with 0.25% trypsin–EDTA. After cell counting, organoids were resuspended in Matrigel and plated at a density of 2000–3000 cells in 10-µL BME2 droplets into 96-well plates. Six days later, the medium was replaced with a drug-supplemented medium. After another 6 days, cell viability was measured using CellTiter-Glo 3D reagent (Promega). Luminescence was measured in a CLARIOstar multimode microplate reader (BMG LABTECH, Germany). The values were normalised to the vehicle.

### Statistical analysis

Data are presented as the mean ± SD of at least three independent experiments or the mean ± SEM value as stated. The normality and homogeneity of variance were evaluated using Graphpad Prism 7.0. Statistical significance was assessed by Student’s *t*-test, ANOVA followed by Dunnett’s or Tukey’s multiple comparisons test or non-parametric test.

## Supplementary information

Supplementary Information

Supplementary Table 1

Supplementary Table 2

Supplementary Table 3

Supplementary Table 4

## Data Availability

The data used during the current study are available from the corresponding author on reasonable request.
