# Supplementary material for: Metformin sensitises hepatocarcinoma cells to methotrexate by targeting dihydrofolate reductase
Source: Cell Death Dis. 2021 Oct 2;12(10):902. doi: 10.1038/s41419-021-04199-1 (PMC8487431; doi:10.1038/s41419-021-04199-1)
Supplement: Supplementary file 1 — Supplementary Information [file 41419_2021_4199_MOESM1_ESM.pdf]

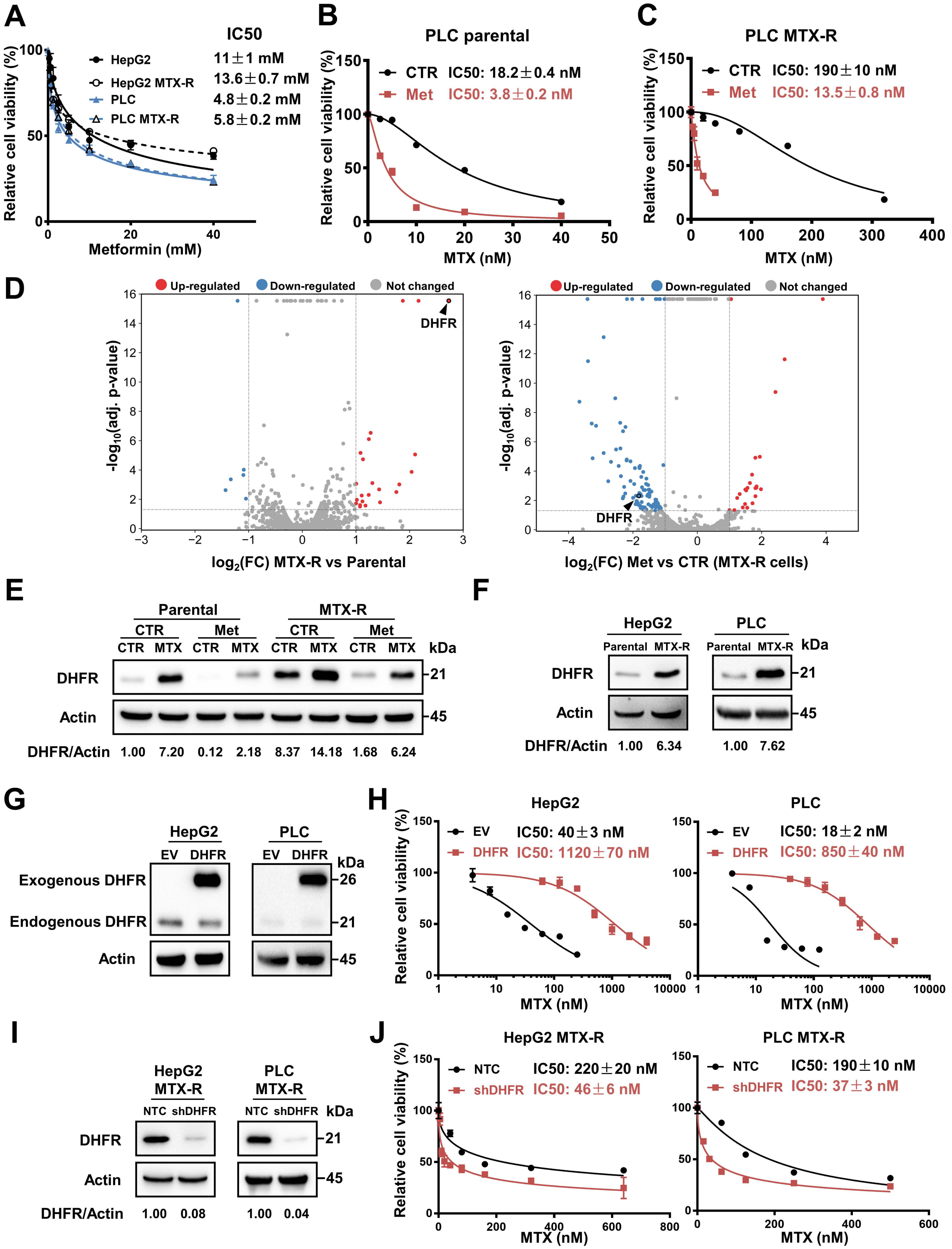

Supplementary Figure 1. Metformin sensitizes hepatocarcinoma cells to MTX treatment by inhibiting DHFR.

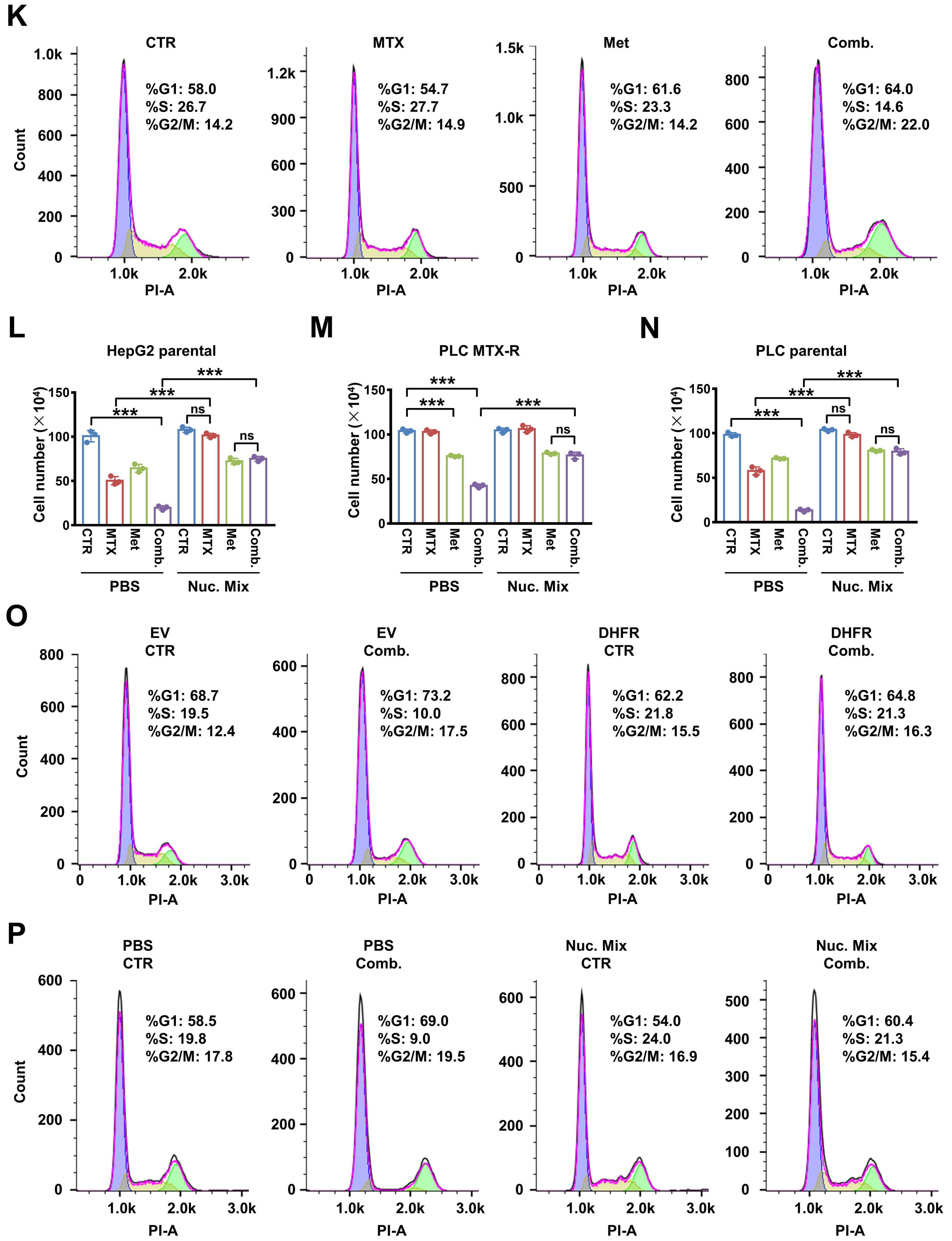

Supplementary Figure 1. Metformin sensitizes hepatocarcinoma cells to MTX treatment by inhibiting DHFR.

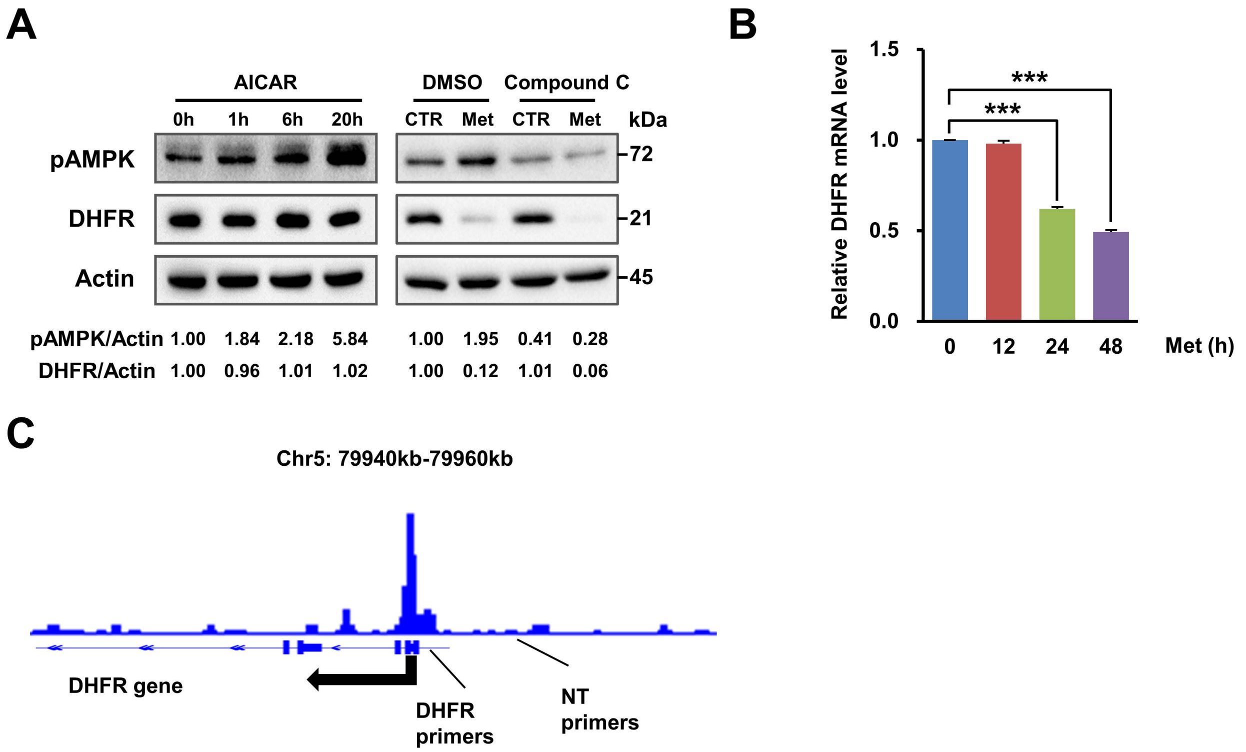

Supplementary Figure 2. Metformin transcriptionally suppresses DHFR via E2F4.

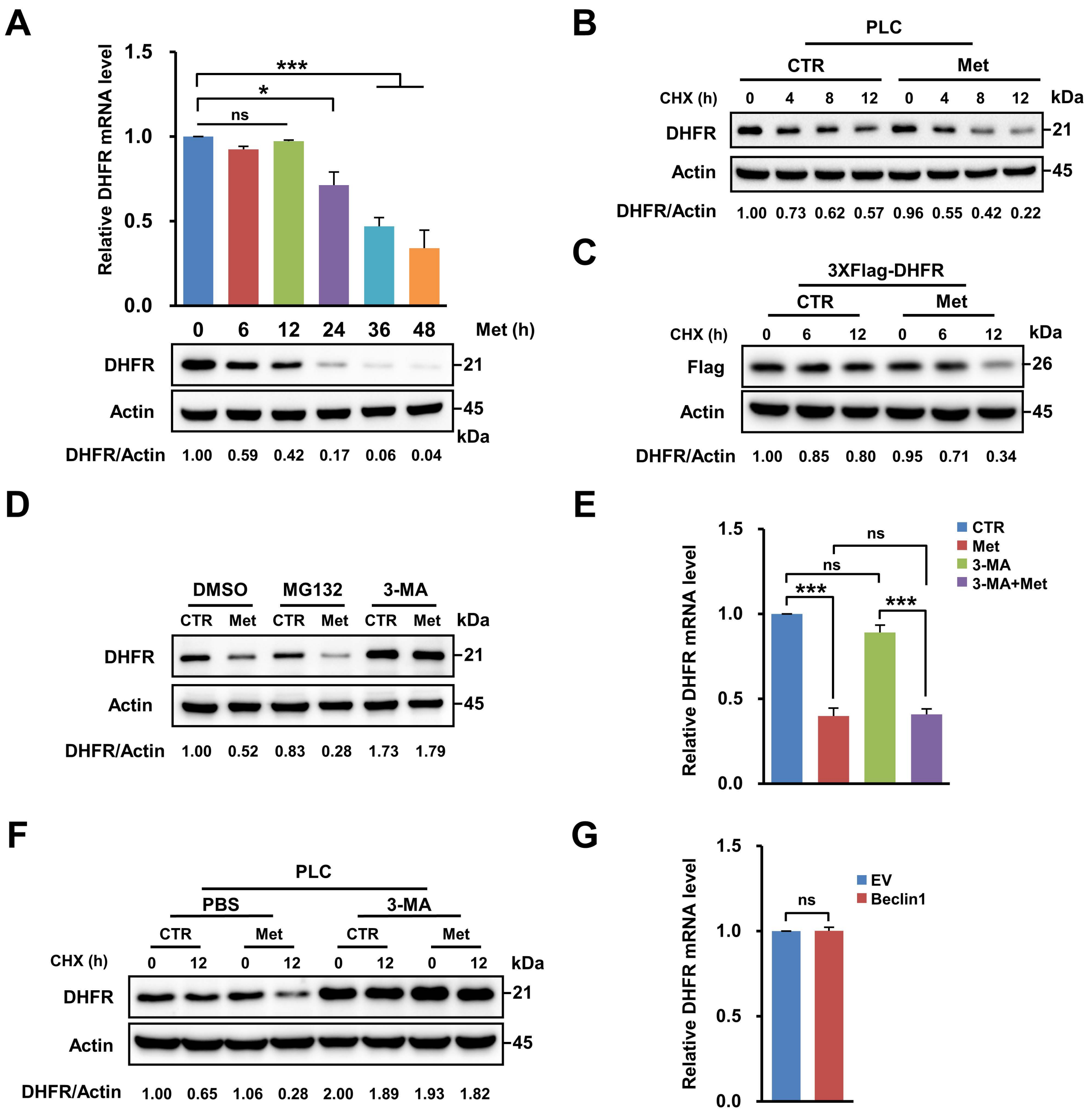

Supplementary Figure 3. Metformin promotes lysosomal degradation of the DHFR protein.

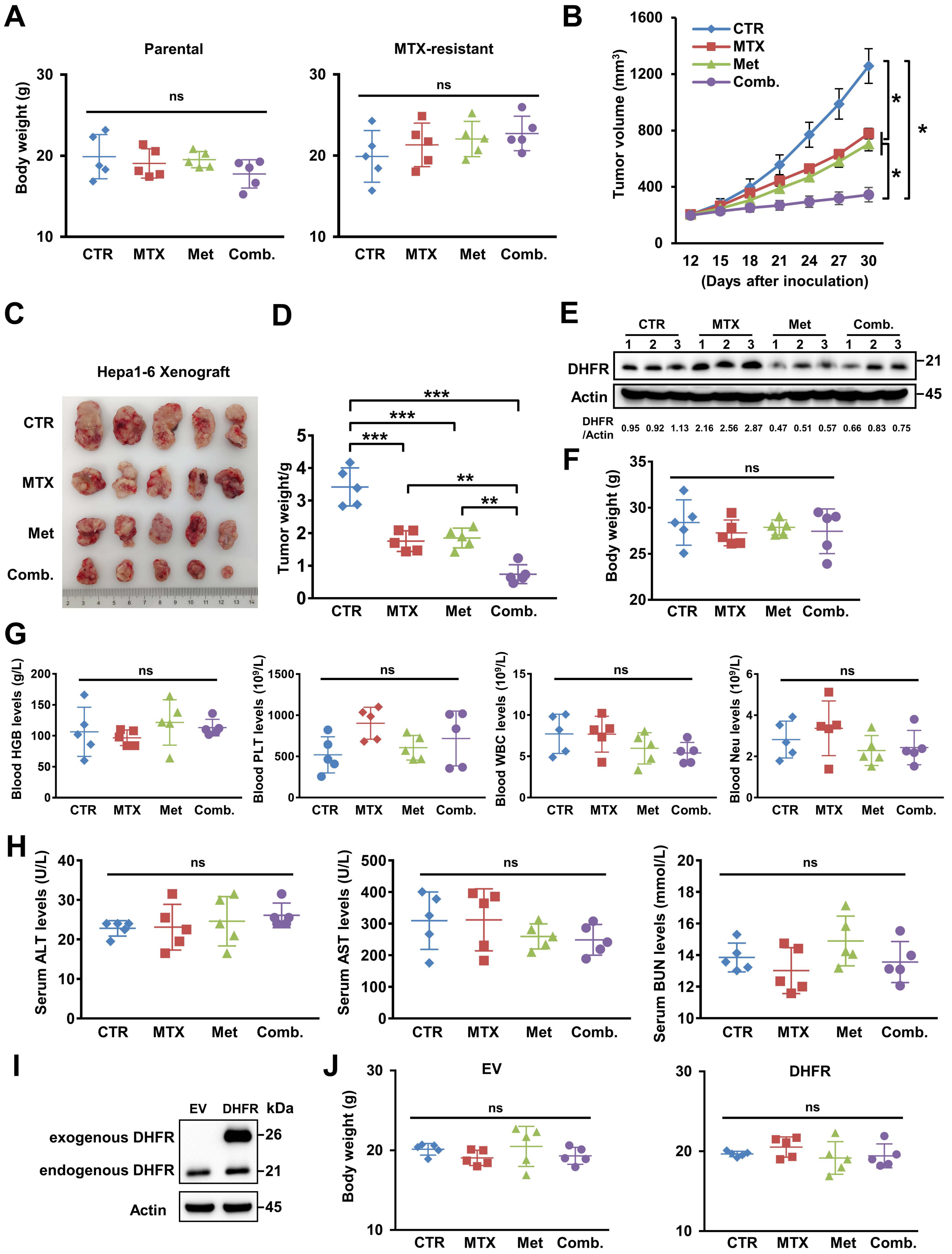

Supplementary Figure 4. DHFR is important for the suppressive effects of metformin on MTX resistance in cancer cells *in vivo*.

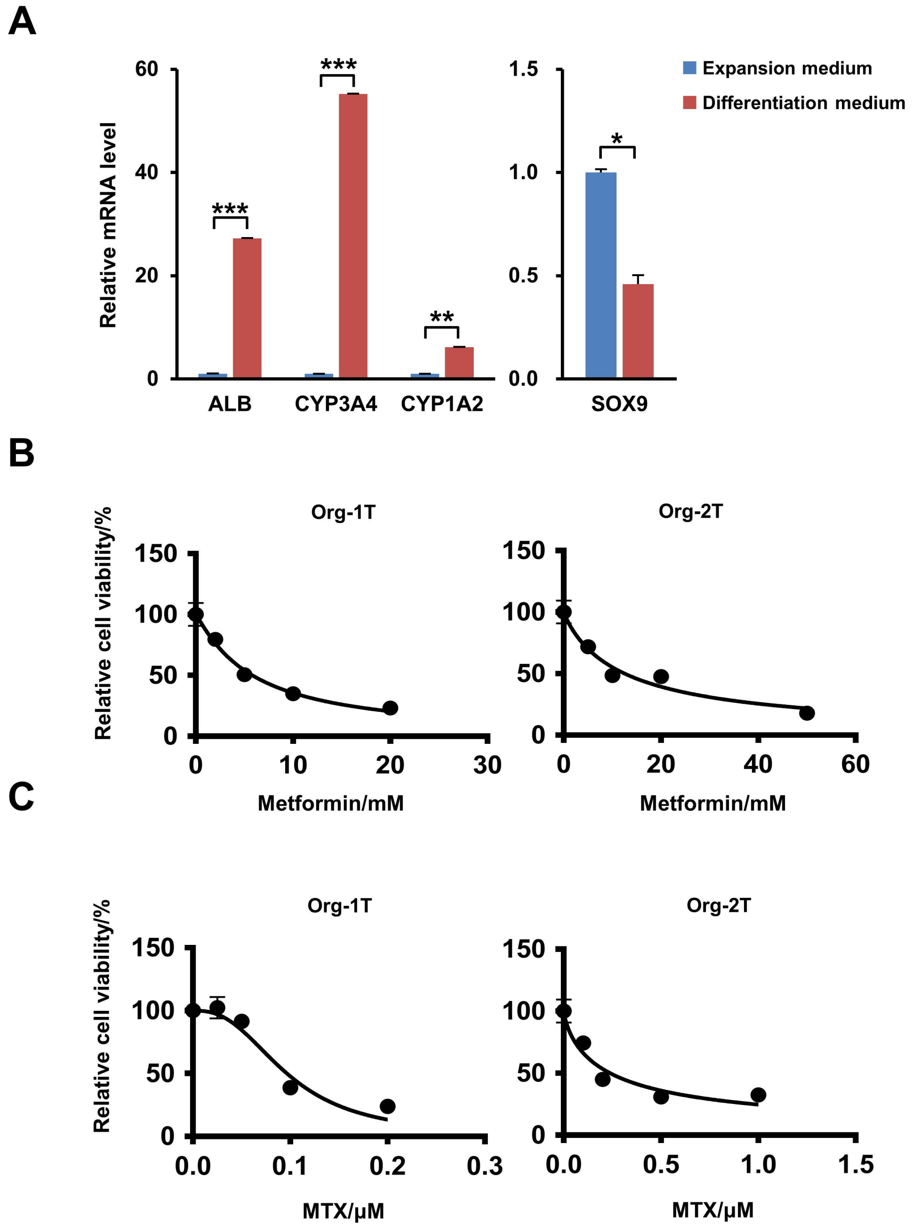

Supplementary Figure 5. Metformin enhances the sensitivity of patient-derived organoids to MTX by decreasing the DHFR level.

Supplementary Figure 1. Metformin sensitizes hepatocarcinoma cells to MTX treatment by inhibiting DHFR.

A, Cell viability of parental or MTX-resistant hepatocarcinoma cells under treatment of different doses of metformin for 72 h was measured by an MTT assay. The IC50 values of metformin in these cells were further calculated with Graphpad Prism 7.0. Data are presented as the mean (±SD) values.

B-C, Cell numbers of parental (B) or MTX-resistant (C) PLC cells under treatment of different doses of MTX for 72 h with or without metformin (1 mM) were measured by a cell counting assay. The IC50 values of MTX in these cells were further calculated with Graphpad Prism 7.0. Data are presented as the mean (±SD) values.

D, Volcano plots comparing MTX-resistant cells to parental cells (left panel) and comparing MTX-resistant cells treated with metformin (2.5 mM) to MTX-resistant cells treated with PBS (right panel) in the proteomic analysis. Proteins with a fold change less than 0.5 (blue) or more than 2 (red) and adj. p-value<0.05 were marked as indicated. The volcano plots were drawn by http://www.bioinformatics.com.cn, an online platform for data analysis and visualization.

E, Western blot analysis of DHFR expression in PLC parental and MTX-resistant cell lines treated with MTX (10 nM), metformin (1 mM) or a combination of both for 48 h.

F, Western blot analysis of DHFR expression in HepG2/PLC parental and MTX-resistant cell lines.

G, Western blot analysis of DHFR expression in HepG2/PLC cell lines expressing 3XFlag-EV or 3XFlag-DHFR.

H, Cell viability of HepG2/PLC cell lines expressing 3XFlag-EV or 3XFlag-DHFR under treatment of different doses of MTX for 72 h was measured by an MTT assay. The IC50 values of MTX in these cells were further calculated with Graphpad Prism 7.0. Data are presented as the mean (±SD) values.

I, Western blot analysis of DHFR expression in HepG2/PLC MTX-resistant cell lines transfected with non-targeting control (NTC) shRNA or DHFR shRNA (shDHFR) virus.

J, Cell viability of HepG2/PLC MTX-resistant cell lines transfected with non-targeting control (NTC) shRNA or DHFR shRNA (shDHFR) virus under treatment of different doses of MTX for 72 h was measured by an MTT assay. The IC50 values of MTX in these cells were further

calculated with Graphpad Prism 7.0. Data are presented as the mean (±SD) values.

K, Representative histogram data showing the distribution of cells in G1, S or G2/M phase in Figure 1H.

L, Parental HepG2 cells were treated with MTX (15 nM), metformin (2.5 mM) or a combination of both metformin with or without the addition of a 25 μM nucleotide mixture for 3 days, and cell numbers in the indicated groups were measured by a cell counting assay.

M-N, MTX-resistant (M) and parental (N) PLC cells were treated with MTX (10 nM), metformin (1 mM) or a combination of both metformin with or without the addition of a 25 μM nucleotide mixture for 3 days, and cell numbers in the indicated groups were measured by a cell counting assay.

O, Representative histogram data showing the distribution of cells in G1, S or G2/M phase in Figure 1K.

P, Representative histogram data showing the distribution of cells in G1, S or G2/M phase in Figure 1L.

Band intensities for protein expressions in the western blot assay were quantitated by ImageJ and normalized to Actin. Data are presented as the mean (±SD) values. Statistical significance was assessed by ANOVA followed by Turkey's multiple comparisons test. *** indicates $P<0.001$ compared between the indicated groups. "ns" indicates no significant difference between the indicated groups.

Supplementary Figure 2. Metformin transcriptionally suppresses DHFR via E2F4.

A, Western blot analysis of the expression of pAMPK and DHFR in PLC cells treated with AICAR (1 mM) for the indicated hours or with DMSO or compound C (10 μM) with or without metformin (1 mM) for 48 h. Band intensities for protein expressions were quantitated by ImageJ and normalized to Actin.

B, qRT-PCR analysis of DHFR mRNA levels in PLC cells treated with metformin (1 mM) for the indicated hours.

C, A diagram showing the positions of the qRT-PCR primers used in the ChIP assay in the DHFR gene. Primers specific to region 3 of the DHFR promoter (DHFR primers) were designed based on analysis of ChIP results in GSM935400. Non-targeting (NT) primers were

designed against sequences in the region distal to the DHFR gene.

Data are presented as the mean (±SEM) or mean (±SD) values. Statistical significance was assessed by ANOVA followed by Dunnett's multiple comparisons test. *** indicates $P<0.001$ compared between the indicated groups.

Supplementary Figure 3. Metformin promotes lysosomal degradation of the DHFR protein.

A, qRT-PCR and western blot analyses of DHFR expression in PLC cells under metformin (1 mM) treatment for the indicated hours.

B, Western blot analysis of DHFR expression in response to cycloheximide (CHX, 1 μg/mL) in PLC cells with or without metformin (1 mM) treatment.

C, Western blot analysis of Flag expression in response to cycloheximide (CHX, 1 μg/mL) in PLC cells expressing 3XFlag-DHFR with or without metformin (1 mM) treatment.

D, PLC cells were treated with PBS, MG132 (5 μM) or 3-MA (1 mM) with or without metformin (1 mM) for 12 h. DHFR protein levels were determined by western blot analysis.

E, HepG2 cells were treated with PBS or 3-MA (1 mM) with or without metformin (2.5 mM) for 24 h. DHFR mRNA levels in the indicated cells were determined by qRT-PCR.

F, PLC cells were pretreated with PBS or 3-MA (1 mM) for 6 h and were then treated with CHX (1 μg/mL), metformin (1 mM) or both for 12 h. DHFR protein levels in the indicated cells were then determined by western blot analysis.

G, DHFR mRNA levels in HepG2 cells expressing 3XFlag-EV or 3XFlag-Beclin1 were determined by qRT-PCR.

Band intensities for protein expressions in the western blot assay were quantitated by ImageJ and normalized to Actin. Data are presented as the mean (±SEM) of three independent experiments. Statistical significance was assessed by Student's t-test or ANOVA followed by Dunnett's or Turkey's multiple comparisons test. * and *** indicate $P<0.05$ and 0.001, respectively, compared between the indicated groups. "ns" indicates no significant difference between the indicated groups.

Supplementary Figure 4. DHFR is important for the suppressive effects of metformin on

MTX resistance in cancer cells *in vivo*.

A, The body weights of mice in the xenograft model described in Figure 4A-4D on day 33 were measured and compared.

B-H, Male C57B6 mice were implanted subcutaneously with Hepa1-6 cells in the flank. After the average tumour volume reached approximately 200 mm^3, mice were treated with an i.p. injection of saline or MTX (10 mg/kg) every four days and intragastric administration of water or metformin (160 mg/kg) every other day (n=5 per group).

B, Tumour volume was determined based on calliper measurements every three days from day 12 to day 30.

C-D, Tumours described in B were excised on day 32. Tumour volume (C) and tumour weight (D) were compared between the indicated groups.

E, Western blot analysis of DHFR expression using lysates of tumour tissues described in B. Actin was used as the loading control.

F, The body weights of mice in the C57B6 xenograft model on day 32 were measured and compared.

G, Contents of hemoglobin (HGB), platelet (PLT), white blood cell (WBC) and neutrophils (Neu) in the blood of indicated groups in the C57B6 xenograft model were measured by a Mindray blood cell analyzer.

H, Enzyme activities of ALT and AST and concentrations of urea nitrogen (BUN) in the serum of indicated groups in the C57B6 xenograft model were measured by a HITACHI biochemical automatic analyzer.

I, Western blot analysis of DHFR expression in MTX-resistant HepG2 EV and DHFR cells.

J, The body weights of mice in the xenograft model described in Figure 4E-4H on day 33 were measured and compared.

Band intensities for protein expressions in the western blot assay were quantitated by ImageJ and normalized to Actin. Data are presented as the mean (±SEM) or mean (±SD) values. Statistical significance was assessed by ANOVA followed by Turkey's multiple comparisons test. *, **, and *** indicate $P<0.05$, 0.01, and 0.001, respectively, compared between the indicated groups. "ns" indicates no significant difference among the indicated groups.

Supplementary Figure 5. Metformin enhances sensitivity to MTX in patient-derived organoids by decreasing the DHFR level.

A, qRT-PCR analysis of the expression of ALB, CYP3A4, CYP1A2 and SOX9 in Org-1N cultured in expansion medium (EM) or differentiation medium (DM). Data are presented as the mean (±SD) of three independent experiments. * indicates $P<0.05$ compared to the EM group. B-C, IC50 values of metformin (B) and MTX (C) in Org-1T and Org-2T were measured using Cell Titer-Glo and analysed by GraphPad Prism 7.0.

Data are presented as the mean (±SD) values. Statistical significance was assessed by Student's t-test. *, **, and *** indicate $P<0.05$, 0.01, and 0.001, respectively, compared between the indicated groups.
